# A Novel Antibody Targeting the Second Extracellular Loop of the Serotonin 5-HT2A Receptor Inhibits Platelet Function

**DOI:** 10.3390/ijms23158794

**Published:** 2022-08-08

**Authors:** Jean E. M. Ramirez, Ahmed B. Alarabi, Fadi T. Khasawneh, Fatima Z. Alshbool

**Affiliations:** 1 Department of Pharmaceutical Sciences, School of Pharmacy, The University of Texas El Paso, El Paso, TX 79902, USA; 2 Department of Pharmacy Practice, Irma Lerma Rangel College of Pharmacy, Texas A&M University, Kingsville, TX 78363, USA; 3 Department of Pharmaceutical Sciences, Irma Lerma Rangel College of Pharmacy, Texas A&M University, Kingsville, TX 78363, USA

**Keywords:** platelet, thrombosis, hemostasis, serotonin, antibody, 5HT_2A_ receptor

## Abstract

Serotonin (5-hydroxytriptamine or 5-HT) is known to be a weak platelet agonist, and is involved in thrombus formation. While 5-HT cannot induce platelet aggregation on its own, when secreted from the alpha granules, it binds to its G-protein Coupled Receptor (GPCR; i.e., 5HT_2A_R), thereby acting to amplify platelet functional responses (e.g., aggregation). Thus, 5HT_2A_R-mediated responses are more involved in the secondary amplification of platelet aggregation in the growing thrombus. Therefore, even though 5-HT can be seen as a weak inducer of platelet activation, it is an important amplifier of aggregation triggered by agonists such as ADP, collagen, and epinephrine, thereby enhancing thrombogenesis. The 5HT_2A_R/5HT_2A_ signaling pathway is of clinical interest to the scientific and medical communities as it has been implicated in the genesis of several forms of cardiovascular disorders. However, efforts to develop antagonists for 5HT_2A_R as therapeutic agents in cardiovascular diseases have thus far failed due to these reagents having deleterious side-effects, and/or to lack of selectivity, amongst other reasons. In light of research efforts that identified that the 5HT_2A_R ligand binding domain resides in the second extracellular loop (EL2; amino acids P^209^-N^233^), we developed an antibody, i.e., referred to as 5HT_2A_RAb, against the EL2 region, and characterized its pharmacological activity in the context of platelets. Thus, we utilized platelets from healthy human donors, as well as C57BL/6J mice (10–12 weeks old) to analyze the inhibitory effects of the 5HT_2A_RAb on platelet activation in vitro, ex vivo, and on thrombogenesis in vivo as well as on 5HT_2A_R ligand binding. Our results indicate that the 5HT_2A_RAb inhibits 5-HT-enhanced platelet activation in vitro and ex vivo, but has no apparent effects on that which is agonist-induced. The 5HT_2A_RAb was also found to prolong the thrombus occlusion time, and it did so without modulating the tail bleeding time, in mice unlike the P2Y12 antagonist clopidogrel and the 5HT_2A_R antagonist ketanserin. Moreover, it was found that the 5HT_2A_RAb does so by directly antagonizing the platelet 5HT_2A_R. Our findings document that the custom-made 5HT_2A_RAb exhibits platelet function blocking activity and protects against thrombogenesis without impairing normal hemostasis.

## 1. Introduction

While platelets are indispensable to physiological hemostasis [1,2,3], when a blood vessel is injured, platelets are able to interact with the unmasked/exposed subendothelial matrix, which leads to platelets adherence and activation [4,5]. Consequently, a number of intraplatelet signaling pathways are triggered, which results in a rise in intracellular calcium levels, synthesis of thromboxane A_2_, and secretion of granule content [2,6] (e.g., ADP and serotonin/5-HT). One important aspect of platelet signaling involves binding of the secreted serotonin (5-HT) to its platelet GPCR, namely the 5HT_2A_ receptor (5HT_2A_R) in order to amplify platelet functional responses (e.g., aggregation) [7]. In this connection, several positive feedback loops including 5-HT secretion are initiated to intensify the extent of aggregation by means of enhancing platelet responsiveness as well as recruiting other platelets to the site of injury, in order to achieve formation of a stable clot [7].

Although platelet activation is a vital component of hemostasis, improper activation and aggregation may lead to thrombosis and thromboembolism [8,9,10,11]. Consequently, antithrombotic drug development efforts have focused on the discovery of reagents/drugs that modulate activation of platelets by their agonists [12,13]. To this end, the serotonin 5HT_2A_R—which couples to Gq [14,15]—is one such target that has been under scrutiny [16]. While 5-HT itself is a weak stimulator of platelet activation, it does however potentiate aggregation triggered by separate agonists, thereby enhancing thrombogenesis [17,18]. The latter notion is supported by clinical data demonstrating that increased blood serotonin levels [19] and hyperactive 5HT_2A_Rs [19] correlate with cardiac events. Another attractive aspect of targeting 5HT_2A_R for therapeutic purposes centers on the notion that its anti-thrombotic activity and bleeding time adverse events can be separated (as demonstrated previously), although the mechanism remains unknown to date [16,20,21]. Moreover, it was found that the expression levels of the 5HT_2A_R increased in patients with coronary thrombosis, and that anti-5HT_2A_R drugs exhibited a clear benefit from a clinical standpoint with regard to protecting against thrombogenesis [22]. To this end, a host of antagonists for 5HT_2A_R were developed throughout the years and investigated for their pharmacological activity [16,20,21,23,24,25]. Unfortunately, none have been approved for clinical use due to significant toxicity and/or lack of selectivity for 5HT_2A_R.

One discernable reason for the failure of 5HT_2A_R antagonism thus far is the fact that these agents were designed empirically without regard to the 5HT_2A_R binding domains information. Therefore, it is critical to understand the structural biology and signaling of 5HT_2A_R. In this connection, mapping of the 5HT_2A_R ligand binding domain revealed that it resides in the second extracellular loop (EL2) of the receptor protein, namely amino acids P^209^-N^233^ [26,27,28].

Based on these considerations, in the present study, we custom-made and characterized the antiplatelet activity of a potentially 5HT_2A_R-function-blocking antibody that targets its ligand binding domain/EL2 region (abbreviated as 5HT_2A_RAb). It is important to note that we have documented success in developing and characterizing similar antibodies designed to target the platelet thromboxane A_2,_ P2Y_1_, and P2Y_12_ receptors [29,30,31]. Our studies revealed that this 5HT_2A_RAb indeed inhibited 5-HT-enhanced ADP-induced human/mice platelet aggregation in vitro, as well as ex vivo in mice. Moreover, 5HT_2A_RAb inhibited 5-HT-enhanced ADP-stimulated platelet secretion, glycoprotein (GP) IIb-IIIa activation, and phosphatidylserine (PS) exposure. Moreover, as for its in vivo activity, mice treated with 5HT_2A_RAb had a significantly prolonged occlusion time, but, unlike the P2Y_12_ antagonist clopidogrel and the 5HT_2A_R antagonist ketanserin, their bleeding time was no different from that of the controls. Interestingly, we also found that serotonin has the capacity to reverse the antithrombotic activity of the 5HT_2A_RAb. Our studies provide insight into the contributions of the EL2 domain to in vivo 5HT_2A_R-dependent platelet activation and the genesis of thrombosis.

## 2. Results

### 2.1. The 5HT_2A_RAb Displaces the 5HT_2A_R Antagonist Ketanserin from Its Binding Sites

**Figure 1:**
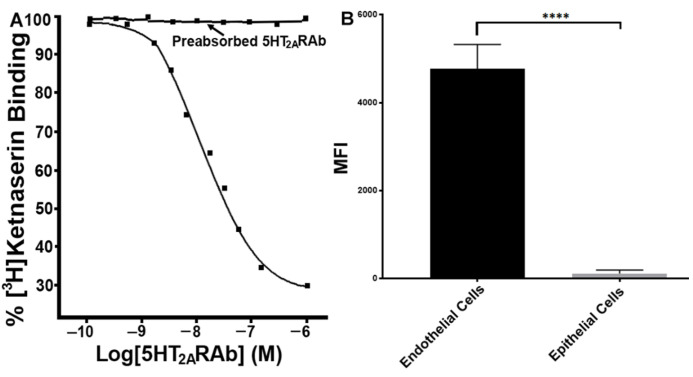
The 5HT_2A_RAb displaces the 5HT_2A_R antagonist [^3^H]ketanserin from its binding sites and binds to endothelial cells but not epithelial cells. (**A**) Binding displacement of the radiolabeled 5HT_2A_R antagonist [^3^H]ketanserin (2 nM) with increasing concentrations of 5HT_2A_RAb (10 nM–1 μM) in human platelets; and preabsorption of 5HT_2A_RAb with its cognate peptide reversed its ability to displace binding of ketanserin from the 5HT_2A_R. (**B**) Flow cytometry binding of the 5HT_2A_RAb to endothelial cells and epithelial cells (**** *p* < 0.0001). These experiments were repeated three times, with blood obtained from three separate healthy human donors, or three separation preparations of endothelial cells and epithelial cells.

While the 5HT_2A_RAb exerts inhibitory effects on serotonin-enhanced platelet function, this is presumed to involve antagonism of the 5HT_2A_R. Indeed, increasing concentrations of the 5HT_2A_RAb (10 nM–1 μM) did displace the radiolabeled 5HT_2A_R antagonist [^3^H]ketanserin (2 nM) from its binding sites on human platelets (Figure 1A). This effect was reversed by preabsorption of 5HT_2A_RAb with its cognate peptide (Figure 1A), which indicates specificity. To further assess its specificity, we also performed binding studies with the 5HT_2A_RAb by employing flow cytometry. Our data show that the 5HT_2A_RAb only interacts with endothelial cells, but not epithelial cells (Figure 1B), as only the former are known to express the 5HT_2A_R protein. These data support the notion that 5HT_2A_RAb’s platelet inhibitory effects involve interaction with the 5HT_2A_R ligand binding domain.

### 2.2. The 5HT_2A_RAb Inhibits Serotonin-Enhanced Human Platelet Aggregation In Vitro

**Figure 2:**
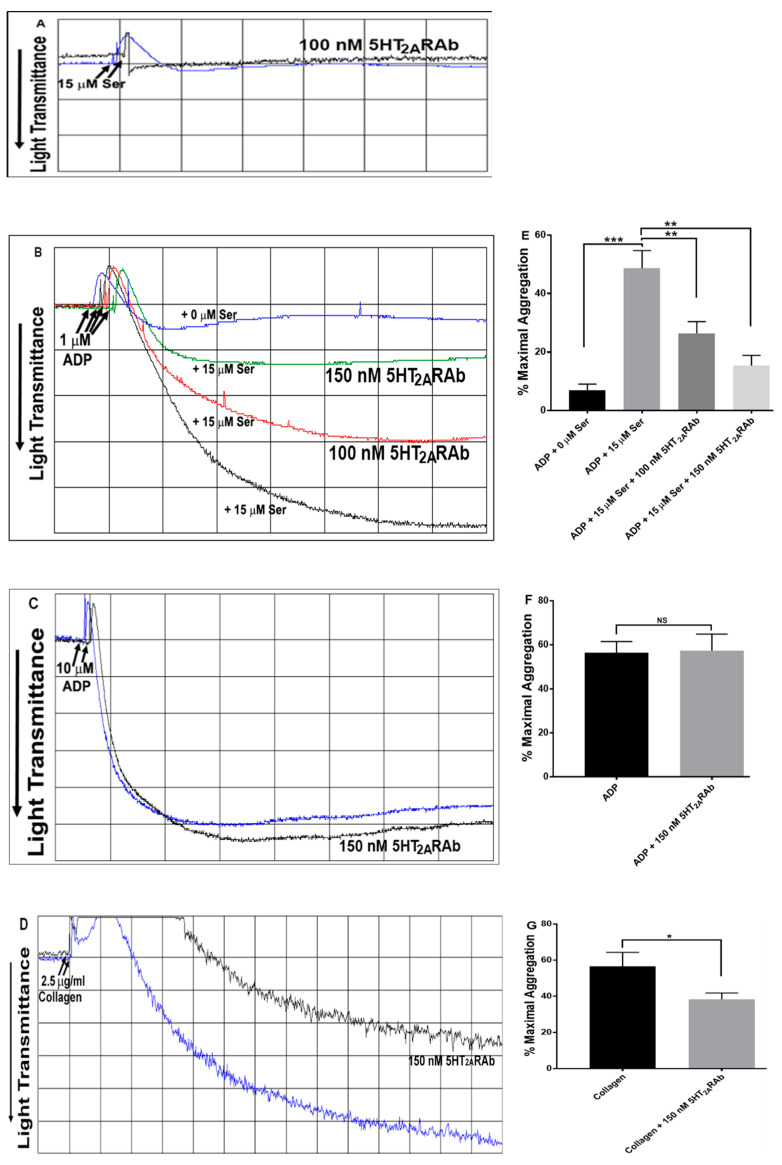
The 5HT_2A_RAb inhibits serotonin-enhanced ADP-induced human platelet aggregation in vitro. (**A**) Human PRP was activated with serotonin (15 μM), with or without pre-incubation for 5 min with the 5HT_2A_RAb (100 nM). (**B**) Human PRP was incubated in the presence and absence of increasing doses of 5HT_2A_RAb (100–150 nM) for 5 min before being activated with submaximal concentration of ADP (1 μM) with or without serotonin (15 μM); (**E**) shows quantification of the maximum aggregation data. (**C**) Human PRP was pre-incubated with 5HT_2A_RAb (150 nM) for 5 min before activation with ADP (10 μM); (**F**) shows quantification of the maximum aggregation data. (**D**) Human PRP was pre-incubated with 5HT_2A_RAb (150 nM) for 5 min before activation with collagen (2.5 μg/mL); (**G**) shows quantification of the maximum aggregation. (* *p* < 0.05; ** *p* < 0.01; *** *p* < 0.001; NS, nonsignificant). Each experiment was repeated three times, with blood obtained from three separate healthy human donors.

Our initial analysis showed that serotonin/5-HT (15 μM) alone does not induce platelet aggregation in human PRP, but rather shape change (Figure 2A). In contrast, we did observe platelet aggregation when stimulating with ADP (1 μM), albeit a weak response given the subthreshold dose we used. Concurrent addition/stimulation with serotonin (15 μM) and ADP (1 μM) on the other hand resulted in a significant platelet aggregation response (Figure 2B). These results demonstrate that serotonin by itself cannot induce platelet aggregation, but that it does have the capacity to enhance ADP-induced aggregation. Given that serotonin produces its effects via the 5HT_2A_R, we investigated if the 5HT_2A_RAb that targets its ligand-binding site would inhibit serotonin-enhanced ADP-induced platelet aggregation. The data showed that the 5HT_2A_RAb did inhibit serotonin-enhanced ADP-induced platelet aggregation dose-dependently (100–150 nM; Figure 2B), when compared to the vehicle control. We next confirmed that 5HT_2A_RAb does not affect the platelet activity in the absence of serotonin, as shown by our results that it did not exert any effects on 10 µM ADP-induced aggregation (Figure 2C). We also examined the effect of the 5HT_2A_RAb on collagen-induced aggregation. Our results show, as was previously documented with small molecule 5HT_2A_R antagonists [32,33,34,35], that the 5HT_2A_RAb did significantly inhibit collagen-induced aggregation (Figure 2D). As one would predict, 15 µM 5-HT-induced platelet shape change was inhibited by the 100 nM of 5HT_2A_RAb (Figure 2A).

### 2.3. The 5HT_2A_RAb Inhibits Serotonin-Enhanced Mouse Platelet Aggregation Ex Vivo

**Figure 3:**
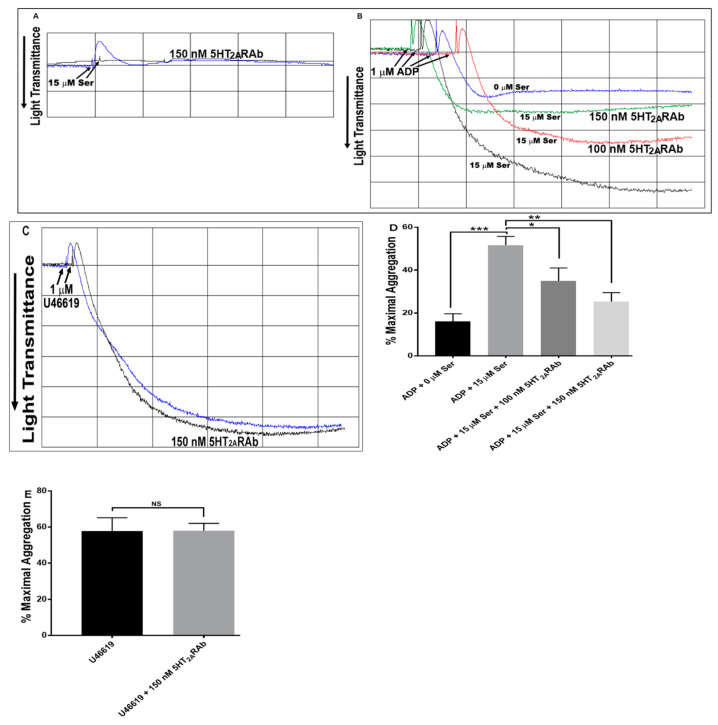
The 5HT_2A_RAb inhibits serotonin-enhanced ADP-induced mouse platelet aggregation ex vivo. Mouse PRP obtained from either vehicle- or 5HT_2A_RAb-injected mice (100–150 nM), once daily for 5 days was stimulated with 15 μM serotonin (**A**), a submaximal concentration of ADP (1 μM) in the presence or absence of 15 μM serotonin (**B)** or with 1 μM U46619 (**C**). (**D**) and (**E**) show quantification of the maximum aggregation data in (**B**) and (**C**) respectively. (* *p* < 0.05; ** *p* < 0.01; *** *p* < 0.001; NS, nonsignificant). Each experiment was repeated three times, with blood pooled from 6–8 mice each time.

We next examined whether the “antagonistic” activity of the 5HT_2A_RAb in the context of aggregation would persist if it was injected into live mice, under multi-dosing conditions. Thus, mice were intravenously injected once daily for 5 days with 5HT_2A_RAb (100 and 150 nM). The results revealed that serotonin-induced platelet shape change was inhibited in the 5HT_2A_RAb (150 nM)-treated mice (Figure 3A). Moreover, 5HT_2A_RAb was found to exert inhibitory effects on serotonin-enhanced ADP-induced aggregation in platelets from the 5HT_2A_RAb injected mice, when compared to the vehicle control (Figure 3B). This finding demonstrates that the inhibitory effects of the 5HT_2A_RAb are maintained under multi-dosing conditions in live animals. It was also found that the 5HT_2A_RAb does not have any apparent effects on the aggregation that is triggered by 1 µM of the thromboxane A_2_ agonist U46619 (Figure 3C).

### 2.4. The 5HT_2A_RAb Inhibits Serotonin-Enhanced Dense Granule Secretion in Human and Mouse Platelets

**Figure 4:**
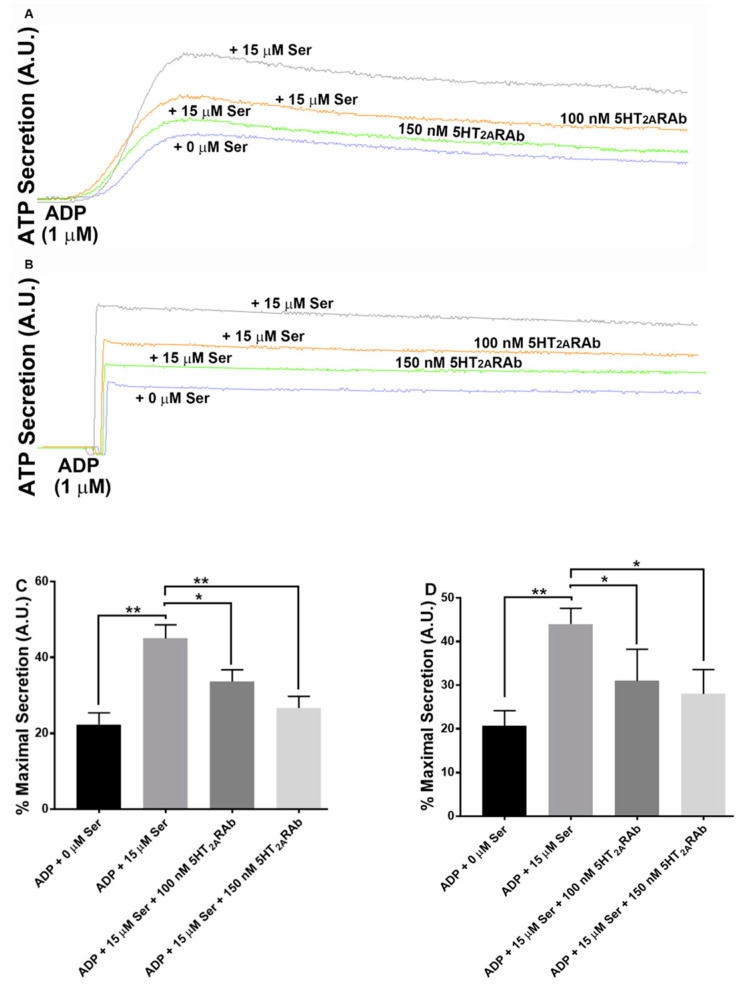
The 5HT_2A_RAb inhibits serotonin-enhanced ADP-induced dense granule secretion in human (in vitro) and mouse platelets (ex vivo). (**A**) Human PRP was incubated with luciferase luciferin (12.5 μL), in the presence and absence of increasing doses of 5HT_2A_RAb (100–150 nM) for 5 min before being activated with a submaximal concentration of ADP (1 μM) with or without serotonin (15 μM); (**C**) shows quantification of the maximum secretion data. (**B**) Mouse PRP obtained from vehicle- or 5HT_2A_RAb-injected mice (100–150 nM) once daily for 5 days, preincubated with luciferase luciferin (12.5 μL), and stimulated with submaximal concentration of ADP (1 μM) in the presence or absence of serotonin (15 μM); (**D**) shows quantification of the maximum secretion data. ATP release (dense granules) was detected as luminescence and measured by a lumi aggregometer. (* *p* < 0.05; ** *p* < 0.01. Each experiment was repeated 3 times, with blood pooled from 6–8 mice each time and data were analyzed using one-way ANOVA.

Agonist-induced granules release plays a very important role in the amplification of initial platelet activation during primary hemostasis [36,37,38]. Accordingly, we determined if the 5HT_2A_RAb exerts any impact on dense granule secretion (ATP). In line with our aggregation data, we observed dose-dependent inhibition of serotonin-enhanced ADP-induced ATP secretion from human platelets treated with 5HT_2A_RAb (100–150 nM; Figure 4A). Similar results were obtained under ex vivo experimental setting, as described before (Figure 4B).

### 2.5. The 5HT_2A_RAb Inhibits Serotonin-Enhanced Platelet Secretion, Glycoprotein IIb-IIIa Activation, and Phosphatidylserine Exposure

**Figure 5:**
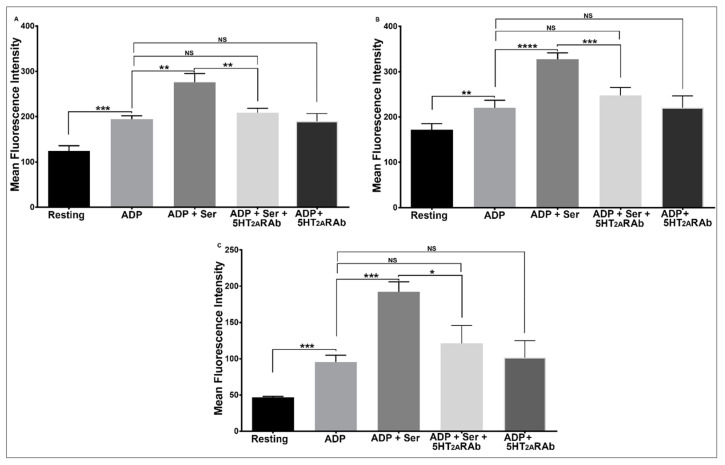
The 5HT_2A_RAb inhibits serotonin-enhanced ADP-induced α granule secretion, GPIIb-IIIa activation, and PS exposure in human platelets in vitro. Washed human platelets were incubated in the presence or absence of the 5HT_2A_RAb (150 nM) for 5 min and then stimulated with ADP (1 μM), in the presence and absence of serotonin (15 μM), for 3 min. The reactions were stopped by fixing the platelets with 2% formaldehyde for 30 min at room temperature. (**A**) Platelets were incubated with FITC-conjugated anti-P-selectin antibody, (**B**) FITC-conjugated PAC-1 antibody, or (**C**) FITC-conjugated Annexin V antibody, and the fluorescence intensity was measured by flow cytometry (* *p* < 0.05; ** *p* < 0.01; *** *p* < 0.001; **** *p* < 0.0001; NS, nonsignificant). Each experiment was repeated three times, with blood obtained from three separate healthy human donors and data were analyzed using one-way ANOVA.

We next examined whether the 5HT_2A_RAb exhibits inhibitory effects on separate platelet functional responses that are also known to be enhanced by serotonin, namely α-granule secretion (P-selectin expression), GPIIb-IIIa activation, and PS exposure. Thus, human platelets were treated with 150 nM 5HT_2A_RAb before being stimulated with ADP (1 µM) alone or with a combination of ADP (1 µM) and serotonin (15 µM). As expected, the 5HT_2A_RAb did reverse the enhanced P-selectin expression, GPIIb-IIIa activation, and PS exposure responses (Figure 5A–C), whereas it had no detectable effects on those responses when induced by ADP alone. Based on these data, we conclude that 5HT_2A_RAb inhibits serotonin-enhanced ADP-induced expression of several markers of platelet activation, in addition to aggregation and dense granule secretion.

### 2.6. The 5HT_2A_RAb Prolongs the Thrombus Occlusion Time but Not the Tail Bleeding Time

**Figure 6:**
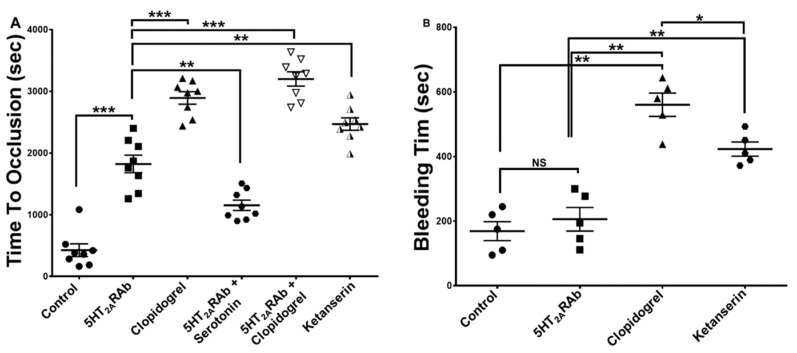
The 5HT_2A_RAb prolongs the time to occlusion but, unlike clopidogrel and ketanserin, does not prolong the tail bleeding time, in mice. Mice were injected via the tail vein with 150 nM 5HT_2A_RAb, once daily for 5 days, 1 mg/kg of the 5HT_2A_R antagonist ketanserin, or 6 mg/kg clopidogrel two hours before being subjected to the FeCl_3_ thrombosis and tail bleeding time models. (**A**) Mean occlusion times for mice treated with vehicle, 150 nM 5HT_2A_RAb, 6 mg/kg clopidogrel, a combination of 50 nM 5HT_2A_RAb and 3 mg/kg clopidogrel, with 150 nM 5HT_2A_RAb followed by 1 mg/kg serotonin, or with 1 mg/kg ketanserin (n = 8 in each case). (**B**) Mean bleeding times for mice treated with vehicle, 150 nM 5HT_2A_RAb, 6 mg/kg clopidogrel, or 1 mg/kg ketanserin (n = 5 in each case). Each point represents the occlusion time or tail bleeding time of a single animal. Data were compared by running the Mann–Whitney test using GraphPad Prism (*** *p* < 0.001; ** *p* < 0.01; * *p* < 0.05; NS, nonsignificant).

We next sought to assess whether the antiplatelet activity 5HT_2A_RAb would hold under in vivo conditions, for the prospective of purposing it as an anti-thrombosis agent. Thus, mice were tail-vein injected with 150 nM of 5HT_2A_RAb for five days before being subjected to the carotid artery FeCl_3_ injury model. Our results showed a significant increase in time to occlusion in the 5HT_2A_RAb-treated mice compared to the vehicle controls (Figure 6A). Rescue experiments were performed to ascertain the contribution of the platelet 5HT_2A_R to the 5HT_2A_RAb-dependent antithrombotic effects. Our results showed that serotonin did in fact significantly reverse the prolongation in the occlusion time induced by 5HT_2A_RAb (Figure 6A). We next tested the effects of 5HT_2A_RAb on hemostasis in order to assess bleeding as a potential adverse effect, which is common to all clinical antithrombotic agents. We were pleasantly surprised that mice injected with 5HT_2A_RAb had a bleeding time that was no different from the controls (Figure 6B). Additionally, we compared the effects of the 5HT_2A_RAb with that of the widely prescribed P2Y_12_ antagonist clopidogrel and with another small molecule 5HT_2A_R antagonist, namely ketanserin. Our results show that while clopidogrel and ketanserin also protected from thrombus formation, they did prolong the tail bleeding time (Figure 6A,B). Finally, we examined the effects of coadministration of the 5HT_2A_RAb with clopidogrel and found the combination of clopidogrel with the 5HT_2A_RAb exerts significant antithrombotic effects at lower doses (Figure 6A). Together, these results support the notion that 5HT_2A_RAb protects/delays thrombus formation, and that it does so without increasing the risk of bleeding, unlike clopidogrel and ketanserin.

### 2.7. The 5HT_2A_RAb Does Not Affect Platelet Number in Mice

Even though not all antibody therapies are known to be associated with thrombocytopenia, we examined whether this side effect would be observed with the 5HT_2A_RAb. Our studies revealed that once daily injections with 150 nM of 5HT_2A_RAb (or vehicle) for 7 (805 ± 66 *×* 10^3^/μL vs. 843 ± 71 × 10^3^/μL, respectively) or 14 days (822 ± 82 × 10^3^/μL vs. 808 ± 85 × 10^3^/μL, respectively) did not appear to have any detectable effects on platelet count when compared to normal/basal platelet counts (813 ± 73 × 10^3^/μL).

## 3. Discussion

According to the National Blood Clot Alliance, it is estimated that 274 people die every day from blood clots [39,40,41,42,43,44]. This means one person dies every 6 min due to a blood clot [39,40,41,42,43,44]. While the current FDA-approved antiplatelet drugs reduce the risk of recurrent heart attacks, stroke, and death, they are associated with serious side effects (e.g., bleeding) which may outweigh their benefits [45,46,47,48,49,50]. Thus, given the limitations of current thromboembolic therapy (e.g., resistance, and bleeding associated with plavix^®^ and/or aspirin), developing safer drugs continues to be a sought-after goal.

In light of the role the 5HT_2A_ signaling pathway plays in the genesis of several forms of cardiovascular disorders, including thrombosis [17,18,19], efforts were undertaken to explore the potential beneficial effects of 5HT_2A_R antagonists as therapeutic agents in patients with cardiovascular disease. Unfortunately, though, currently there are no FDA-approved 5HT_2A_R antagonists available for managing thrombotic disease. Nonetheless, a host of such antagonists were designed and examined throughout the years [16,20,21,23,24]. For example, the 5HT_2A_R antagonist ketanserin was shown in clinical studies to reduce the incidence of myocardial infarction in patients with coronary artery stenosis [23]. However, these results were accompanied by deleterious side-effects due to lack of selectivity for 5HT_2A_R [23]. Subsequent attempts to design antagonists with improved selectivity profiles produced sarpogrelate, which (initially) showed promise in human clinical studies [24,51]. However, this drug did not receive approval by the United States FDA due to limitations that are likely associated with its activity on 5HT_2B_R. Later, an experimental 5HT_2A_R antagonist, namely AR246686, was synthesized and tested for its selectivity and efficacy [16]. While AR246686 lacked affinity toward 5HT_2B_R and 5HT_2c_R, and exhibited antithrombotic effects in vitro and in vivo, its significant toxicity prevented its clinical utility. It is to be noted that the empirical design of these agents and the lack of understanding of the 5HT_2A_R binding domains likely contributed to this failure.

With regard to the 5HT_2A_R ligand interaction sites, it has been shown that its ligand binding domain resides in the second extracellular loop (EL2; P^209^–N^233^) [26,27,28]. Furthermore, this site was found to contain several amino acids (S^219^, L^229^, A^230^, and N^233^) coordination sites [26,27,52,53,54], which are either shared or unshared between a number of ligands. Based on this consideration, in our efforts to develop a 5HT_2A_R antagonist, we custom made and characterized an antibody that targets its ligand binding pocket, i.e., the second extracellular loop (designated 5HT_2A_RAb) for its function-blocking activity, in platelets. We demonstrated that the antibody developed against the ligand binding domain of 5HT_2A_R has antiplatelet activities in both human and murine platelet models. Thus, we showed that the 5HT_2A_RAb inhibited serotonin-enhanced agonist-induced platelet aggregation, whereas it exerted no effects on agonist (alone)-induced aggregation, not only in vitro but also ex vivo (under multi-dosing conditions). The latter observation is contrary to clopidogrel (Plavix^®^), which is known to inhibit non-ADP pathways [55,56]. Of note however, the 5HT_2A_RAb did impair collagen-dependent aggregation, which was also found to be the case with small molecule inhibitors of the 5HT_2A_R [32,33,34,35]. This observation is consistent with the critical role serotonin plays in the collagen-dependent aggregation response [32,33,34,35]. Moreover, the 5HT_2A_RAb was also found to inhibit the ability of serotonin to enhance agonist-induced secretion, GPIIb/IIIa activation, and PS exposure. Furthermore, we also found that the 5HT_2A_RAb not only protected against thrombosis but that—unlike the P2Y_12_ antagonist clopidogrel—it did so without interfering with physiological hemostasis, in mice. Consistent with this finding, it was previously suggested that targeting 5HT_2A_R allows for the separation of anti-thrombotic activity and bleeding [16,20,21]. Aside from findings that 5-HT alone does not stimulate procoagulant responses [57], a likely explanation derives from the belief that the 5HT_2A_R-mediated responses are more involved in the secondary amplification of platelet aggregation in the growing thrombus, rather than playing a primary role in formation and stabilization of a hemostatic thrombus, such as ADP and thromboxane A_2_. Another explanation is that the antibody cannot penetrate the endothelial cell layer, and hence will not reach the smooth muscle cells; thus, this approach allows for the selective modulation of the platelet 5HT_2A_R, but not those of the smooth muscle, which are known to affect bleeding time. Notably, and in-line with this notion, we previously reported that an antibody targeting the ligand-binding domain of the human platelet thromboxane A_2_ receptor protein exhibited a similar pharmacological profile, namely protection from thrombus formation without impairing hemostasis [29]. Of note, the 5HT_2A_R antagonist ketanserin was found to modulate hemostasis, contrary to the 5HT_2A_RAb, which suggests that this antibody may be safer than some of the small molecule antagonists for this receptor. It is also to be noted that one cannot exclude contributions from non-platelet 5HT_2A_Rs to the 5HT_2A_RAb-dependent antithrombotic effects. Hence, to address this issue we performed rescue experiments with serotonin, which showed significant reversal of the 5HT_2A_RAb effects. It is probable that the 5HT_2A_RAb may not be used alone, but rather in combination with other antiplatelet agents. This notion was supported by the finding that coadministration of the 5HT_2A_RAb with clopidogrel, produced significant antithrombotic effects at lower doses. At last but not the least, radioligand binding studies revealed that the aforementioned 5HT_2A_RAb inhibitory effects appear to derive from its ability to interact with the 5HT_2A_R protein. Moreover, separate binding studies revealed that the 5HT_2A_RAb interacts with endothelial cells, but not epithelial cells, which further supports its specificity, as only the former are known to express the 5HT_2A_R protein. Together, these data support the notion that the 5HT_2A_RAb would be superior to classical small molecule-based antagonists because its effects would be limited to the platelet 5HT_2A_Rs. It is noteworthy that an antibody-based therapy is widely utilized in many disease states including those that are thrombosis-based. For example, the antibody against the GPIIb/IIIa, known as abciximab/ReoPro^®^, has previously been approved by the FDA as an antiplatelet agent, despite the fact that the primary risk associated with its use is bleeding [58,59,60]. Thus, it is possible that the 5HT_2A_RAb would also have potential clinical use. Finally, efforts to developed anti-5HT_2A_R small molecule inhibitors are continuing to date [35], thereby validating the 5HT_2A_R as an attractive therapeutic target [22,61].

## 4. Methods and Materials

### 4.1. Reagents and Materials

Serotonin hydrochloride, ADP, U46619, clopidogrel bisulfate, and ketanserin (+)-tartrate salt were obtained from Sigma Aldrich (St. Louis, MO, USA). Collagen, stir bars, and other disposables were from Chrono-Log (Havertown, PA, USA). FITC-conjugated Annexin V, anti-P-selectin, and PAC-1 antibodies were purchased from Cell Signaling Technology, Inc. (Danvers, MA, USA). The 5HT_2A_ receptor targeting polyclonal antibody, designated 5HT_2A_RAb, was custom made (against the Q^216^-N^233^ region of EL2 of the human protein) and antigen-affinity purified by a commercial vendor (Abbiotec Inc., San Diego, CA, USA). Of note, while the mouse and human 5HT_2A_R receptors are identical in 19 of the 25 amino acids of EL2, all ligand recognition sites reported thus far appear to be conserved between the two species.

### 4.2. Animals

C57BL/6 mice were obtained from Jackson Laboratory (Bar Harbor, ME, USA). Mice were housed in groups of 1–4 at 24 °C, under 12/12 light/dark cycles, with access to water and food ad libitum. All experiments involving animals were performed in compliance with the institutional guidelines, and were approved by the Institutional Animal Care and Use Committee.

### 4.3. Human and Murine Platelet Preparation

Blood was drawn from healthy volunteers who self-reported not taking any medication for at least 1 week prior to collection, or from C57BL/6 mice (10–12 weeks old). Mice were anesthetized and blood was collected from the heart. Coagulation was inhibited by 3.8% *w*/*v* sodium citrate solution (1 part sodium citrate to 9 parts blood). Human or mouse platelet rich plasma (PRP) was obtained by centrifugation at room temperature, as previously described [29,30,31]. Platelets were counted with automated hematology analyzer and their count adjusted to 7 × 10^7^ platelets/mL, prior to each experiment. Washed human platelets were prepared as we described before [30,31]. PRP was isolated in the presence of 0.37 units/mL apyrase and 10 ng/mL PGI_2_ by centrifugation at 150× *g* for 10 min at 20 °C. Next, PRP was centrifuged at 900× *g* for 10 min, and pelleted platelets were resuspended in HEPES/Tyrode’s buffer (20 mMHEPES/NaOH, pH 6.5, 128 mM NaCl, 2.8 mM KCl, 1 mM MgCl_2_, 5 mM D-glucose, 12 mM NaHCO_3_, 0.4 mM NaH_2_PO_4_) containing 1 mM EGTA, 0.37 units/mL apyrase, and 10 ng/mL PGI_2_. Platelets were washed and resuspended in HEPES/Tyrode’s buffer (pH 7.4) without EGTA, apyrase, or PGI_2_. The final platelet counts were adjusted to 2 × 10^8^ platelets/mL.

### 4.4. In Vitro Platelet Aggregation and ATP Secretion

Human PRP was incubated with the 5HT_2A_RAb (100–150 nM) or without (vehicle; water) for 5 min prior to experiments, as applicable. Platelets were then activated with serotonin alone (15 μM); submaximal concentration of ADP (1 μM), in the presence or absence of 15 μM serotonin; as well as with collagen (2.5 μg/mL). Platelet aggregation was measured by the turbidometric method using a model 700 aggregometer (Chrono-Log Corporation, Havertown, PA, USA). ATP secretion was measured as previously described. Aggregation is also measured in platelets stimulated with serotonin (15 μM) alone or ADP (10 μM) as control with and without pre-incubation with 5HT_2A_RAb. Each experiment was repeated at least 3 times, with blood collected from three different human donors.

### 4.5. Ex Vivo Platelet Aggregation and ATP Secretion

Mice were injected with the 5HT_2A_RAb (100–150 nM) or vehicle/water control using the tail vein (IV) route once daily for 5 days in an attempt to mimic chronic administration of these drugs in patients. Mice were sacrificed 2 h post last injection, and their blood collected. Platelets (with counts adjusted as described before) were stimulated with 1 μM ADP in the presence or absence of 15 μM serotonin, and platelet aggregation and ATP secretion were measured as previously described. Aggregation was also measured in platelets stimulated with serotonin (15 μM) alone or U46619 (1 μM) as control. Each experiment was repeated at least 3 times, with blood pooled from 6-8 mice each time.

### 4.6. Flow Cytometric Analysis

Human washed platelets (2 × 10^8^) were incubated in the presence or absence of 5HT_2A_RAb (150 nM) for 5 min. Platelets were stimulated with 1 μM ADP in the presence or absence of 15 μM serotonin. The reactions were stopped by fixing the platelets with 2% formaldehyde for 30 min at room temperature. Next, platelets were incubated with FITC-conjugated Annexin V, anti-P-selectin, or PAC-1 antibodies at room temperature for 30 min in the dark. Finally, the platelets were diluted 2.5-fold with HEPES/Tyrode buffer (pH 7.4) before the samples were transferred to FACStubes and fluorescence intensities were measured using a BD Accuri C6 flow cytometer and analyzed using CFlow Plus (BD Biosciences, Franklin Lakes, NJ, USA). We also performed flow cytometry-based binding studies with the 5HT_2A_RAb using endothelial and epithelial cells. Data were compared by running one-way ANOVA followed by Tukey’s multiple comparisons test.

### 4.7. In Vivo Ferric Chloride Carotid Artery Injury–Induced Thrombosis Model

These studies were performed as described previously [30,31]. Briefly, mice 10–12 weeks old received injections via the tail vein/IV with the 5HT_2A_RAb (150 nM) or vehicle/water once daily for a period of 5 days, the 5HT_2A_R antagonist ketanserin (1 mg/kg) or the P2Y_12_ antagonist clopidogrel (6 mg/kg) 2 h before being anesthetized with an intraperitoneal (IP) injection of tribromoethanol. Then, the left carotid artery was surgically exposed and cleaned, and baseline carotid artery blood flow was measured by placing a Transonic micro-flowprobe (0.5 mm, Transonic Systems Inc., Ithaca, NY, USA) on the surface of the artery. After stabilization of blood flow, the NaCl solution was removed and 7.5% ferric chloride (FeCl_3_) was applied to a filter paper disc (1-mm diameter) that was immediately placed on top of the artery for 3 min. After 3 min, the filter paper was removed, saline solution was placed in the wound, and carotid blood flow was monitored using a perivascular flowmeter. Blood flow was continuously monitored for 30 min, or until blood flow reached stable occlusion (zero blood flow for 2 min). Data were recorded as the time to vessel occlusion and calculated as the difference in time between stable occlusion and removal of the filter paper containing ferric chloride and compared by running the Mann–Whitney test using GraphPad Prism. Of note, rescue experiments were performed as described above but after intraperitoneal (IP) injections of serotonin (1 mg/kg) prior to the surgical procedure. The serotonin dose was selected based on previous studies [62,63]. Moreover, coadministration studies were also performed in a similar manner, but after mice were injected with 3 mg/kg of clopidogrel and 50 nM of the 5HT_2A_RAb.

### 4.8. Tail Bleeding Time

Mice were tail-vein injected with the 5HT_2A_RAb (150 nM) or vehicle once daily for 5 days, or with the 5HT_2A_R antagonist ketanserin (1 mg/kg), or the P2Y_12_ antagonist clopidogrel (6 mg/kg) 2 h, before their hemostasis response was examined using the tail transection technique [30,31]. Briefly, mice were anesthetized with isoflurane and placed on a 37 °C homeothermic blanket. The distal portion of the tail (5 mm) was transected from the tip using a sterile scalpel. After transection, the tail was immediately immersed in 0.15 M sodium chloride (37 °C, constant temperature) and the time to bleeding cessation was measured. Cessation of bleeding was defined as stoppage of blood loss for at least one minute. If bleeding did not stop within 10 min, pressure was applied to the tail to seal the wound, thus avoiding excessive loss of blood, with the 10 min indicated for statistical analysis purposes; the Mann–Whitney test (GraphPad Prism) was used to analyze the data.

### 4.9. Radioligand Binding Displacement

Resuspended platelets (1 × 10^9^ platelets/mL) were prepared as previously described [64,65,66], before they were incubated with 2 nM [^3^H]ketanserin at room temperature for 10 min. Next, increasing concentrations of the displacing 5HT_2A_RAb (10 nM–1 μM) were added for an additional 45 min. Next, the [^3^H]ketanserin bound platelets were captured by running through 0.45 micron Millipore filters over a vacuum suction unit. The filters were then washed once and counted for radioactivity in a Beckman LS 6000 liquid scintillation counter. To calculate the non-specific binding, the same concentration of radio ligand was competed against 1000-fold excess of unlabeled ketanserin.

### 4.10. Platelet Count

Platelet count was determined in mice injected daily with 150 nM of 5HT_2A_RAb or vehicle for 7 or 14 days using an automated hematology analyzer (Drew Scientific, Miami Lakes, FL, USA).

### 4.11. Statistical Analysis

Data analysis was achieved using the statistical software GraphPad PRISM (San Diego, CA, USA). Results were presented as mean ± SEM or SD. Statistical significance was accepted at *p* < 0.05. Please refer to the figure legends for additional details regarding the specific statistical analysis tests employed for each of our experiments. 

## 5. Conclusions

In conclusion, our study constitutes the first investigation of a function blocking antibody against the 5HT_2A_R and supports the notion that the EL2 region plays a critical role in its structural biology. Moreover, it provides novel information concerning a potential target site, i.e., EL2, for therapeutic intervention. Specifically, the 5HT_2A_RAb produced significant effects on platelet function and protected against thrombosis with no apparent effect on bleeding. Collectively, these data suggest that targeting EL2 of 5HT_2A_R, for which currently there are no interventions available for clinical use, may define a new class of antiplatelet GPCR drugs, and could have widespread therapeutic (and experimental) applications, especially given the limitations of current thromboembolic therapy. Thus, future efforts will focus on generating a monoclonal version of the 5HT_2A_RAb and a pharmaceutical formulation for clinical testing. Finally, these results may aid molecular modeling prediction studies for the design of organic derivatives/agents targeting the 5HT_2A_R.

## Data Availability

Data will be made available by the corresponding author upon request.
